# Office Notes

**Published:** 1871-03

**Authors:** W. H. Shadoan


### OFFICE NOTES.

BY W. H. SHADOAN, D. D. S.

[Continued from Page 63.]

Case 2. Mrs. H. Second right inferior molar. This was one of those cases that we always dislike. The tooth had three large cavities, situated one in the crown, one approximal, another in the buccal surface, all very large and extending into the crown cavity. The approximal extended well down to the gum, the buccal within half a line of the gum. The decay was removed, and the buccal cavity opened into the crown with a small bit of file held in the bayonet file carrier. The dentine had so far been removed between the approximal and the crown cavities, as to need nothing but to chisel away the sharp thin portions of dentine that separated them, and the cavities were ready for the filling.

The rubber dam was placed on the tooth and the one anterior to it, and a cork one inch long placed between the teeth on the opposite side of the mouth, the upper edges of the dam were held up out of the way by the Coggswell clamp, and a slide clamp adjusted to the lower edge of the dam, to act as a weight to keep it out of the way during the operation.

*Method of filling.* First, the filling was introduced into the pits in the floor of the approximal cavity, as in case of one reported in December number of this journal, and when the filling had progressed to the floor of the crown cavity, it was stopped in this cavity, then proceeded to fill the buccal cavity in the same way, using the same No. of gold, and the same instruments as in case one. The point of opening into the crown cavity from the buccal cavity was not so low as in the approximal, so that when the buccal cavity was filled to the point where it opens into the crown cavity, the filling would necessarily be above the bottom of that cavity.

When it reaches that point then the crown and approximal cavities were filled as one cavity, until they reach the level of the gold in the buccal cavity, and then the gold is carried over into it and all are filled as one. The instruments used in this case are for the formation of the fillings, the same as for cases one and two, after that for the remainder of the filling I usually use Nos. 13, 14 and 15, using an instrument of my own make, somewhat like No. 1, of Dr. Butler's pattern, but having a point three times as wide. For condensing the surface of the approximal and buccal fillings, No. 10 is well adapted. I used Nos. 60 and 80 gold for filling this tooth, except in the bottom which was filled with No. 4. In all such cases the cusp that is isolated by the approximal and buccal cavity should be dressed off, so as to allow the gold to overlap it, unless it is very firm and solid, in which case it may be left standing. There is room to display skill in cases like this, although it is called a simple case.

[To be continued.]

